# The Multifunctional Sactipeptide Ruminococcin C1 Displays Potent Antibacterial Activity In Vivo as Well as Other Beneficial Properties for Human Health

**DOI:** 10.3390/ijms22063253

**Published:** 2021-03-23

**Authors:** Clarisse Roblin, Steve Chiumento, Cédric Jacqueline, Eric Pinloche, Cendrine Nicoletti, Hamza Olleik, Elise Courvoisier-Dezord, Agnès Amouric, Christian Basset, Louis Dru, Marie Ollivier, Aurélie Bogey-Lambert, Nicolas Vidal, Mohamed Atta, Marc Maresca, Estelle Devillard, Victor Duarte, Josette Perrier, Mickael Lafond

**Affiliations:** 1 CNRS, Aix-Marseille University, Centrale Marseille, iSm2, 13013 Marseille, France; clarisse.roblin@univ-amu.fr (C.R.); cendrine.nicoletti@univ-amu.fr (C.N.); hamza.olleik@live.com (H.O.); elise.courvoisier-dezord@univ-amu.fr (E.C.-D.); agnes.amouric@univ-amu.fr (A.A.); dru.louis.1@gmail.com (L.D.); m.maresca@univ-amu.fr (M.M.); josette.perrier@univ-amu.fr (J.P.); 2 Centre d’Expertise et de Recherche en Nutrition, ADISSEO France SAS, 03600 Commentry, France; Eric.Pinloche@adisseo.com (E.P.); estelle.devillard@adisseo.com (E.D.); 3 University Grenoble Alpes, CEA, IRIG, CBM, CNRS UMR5249, 38054 Grenoble, France; stevechiumento@gmail.com (S.C.); Christian.BASSET@cea.fr (C.B.); mohamed.atta@cea.fr (M.A.); victor.duarte@cea.fr (V.D.); 4 EA3826, IRS2 Nantes-Biotech, Université de Nantes, 44200 Nantes, France; cedric.jacqueline@univ-nantes.fr; 5 BioAzur Biogroup-Vet’Analys Laboratory, 83400 Hyères, France; marie.masson@univ-tln.fr (M.O.); vetanalys@gmail.com (A.B.-L.); 6 Yelen Analytics, Aix-Marseille University ICR, 13013 Marseille, France; yeleniko@yahoo.fr

**Keywords:** antibiotics, antimicrobial resistance, RiPP, sactipeptide, *Ruminococcus gnavus* E1, peritonitis infection, microbiome, *Clostridium perfringens*

## Abstract

The world is on the verge of a major antibiotic crisis as the emergence of resistant bacteria is increasing, and very few novel molecules have been discovered since the 1960s. In this context, scientists have been exploring alternatives to conventional antibiotics, such as ribosomally synthesized and post-translationally modified peptides (RiPPs). Interestingly, the highly potent in vitro antibacterial activity and safety of ruminococcin C1, a recently discovered RiPP belonging to the sactipeptide subclass, has been demonstrated. The present results show that ruminococcin C1 is efficient at curing infection and at protecting challenged mice from *Clostridium perfringens* with a lower dose than the conventional antibiotic vancomycin. Moreover, antimicrobial peptide (AMP) is also effective against this pathogen in the complex microbial community of the gut environment, with a selective impact on a few bacterial genera, while maintaining a global homeostasis of the microbiome. In addition, ruminococcin C1 exhibits other biological activities that could be beneficial for human health, as well as other fields of applications. Overall, this study, by using an in vivo infection approach, confirms the antimicrobial clinical potential and highlights the multiple functional properties of ruminococcin C1, thus extending its therapeutic interest.

## 1. Introduction

According to the World Health Organization, antibiotic resistance is one of the biggest threats to public health and food safety worldwide. Development of resistance is promoted by exposure to antibiotics. Indeed, antibiotic exposure creates a selective pressure in which bacteria with an acquired or intrinsic resistance have an advantage for survival and for spreading on susceptible bacteria [1,2,3]. As most antibiotics come from natural origins, and, in particular, from microorganisms, bacteria have been exposed to them and started developing resistance long before their discovery by humans [4]. Furthermore, the industrialization of antibiotics has accelerated this phenomenon, and almost all introductions of new antibiotics in clinics has led to the emergence of resistant bacteria within a couple of years, or even in the same year, although some exceptions remain (e.g., vancomycin). Some bacteria have evolved so much over the last few decades that they have become resistant to several classes of antibiotics, or even to all of them. These are designated as multidrug- and pan-drug-resistant (MDR and PDR) bacteria, respectively [5]. Currently, in the United States, approximately 100,000 deaths are caused each year due to antibiotic-resistant pathogens associated with hospital-acquired infections [6]. It is believed that this number will drastically increase up to 10 million worldwide by 2050, making antimicrobial resistance the leading cause of death worldwide [7]. Moreover, the total economic loss accredited to antibiotic resistance in the US is estimated to be $55 billion per year [6]. Effective antibiotics are needed not only for the treatment of reported infections, but also for many medical procedures such as common surgeries that could be lethal in the case of postoperative infections [8]. Furthermore, procedures that require or provoke the depression of the immune system, such as organ transplantation or chemotherapy, could be too dangerous to perform if no effective antibiotics are available. As a result, the rise of resistance could lead back to the “dark ages of medicine”, which refers to the era before the discovery of antibiotics. As only two classes of antibiotics, the lipopeptides and the diarrylquinolines, have been discovered since the 1960s [9,10], there is an urgent need to identify new compounds that could reach the clinic to fight MDR or PDR bacteria.

In this context, several alternatives to antibiotic treatment such as phage therapy, immunotherapy, or microbiota transplantation are under investigation to overcome the antibiotic resistance crisis [11]. Among these alternatives, antimicrobial peptides (AMPs) from the ribosomally synthesized and post-translationally modified peptide (RiPP) family constitute a potential trove of active molecules. RiPPs are produced by organisms from the three domains of life (archaea, prokaryotes, and eukaryotes) and exert multiple types of biological activities, including antibacterial and antimicrobial activities, as well as insecticidal, nematoxic, or anti-cancer effects, among others. Although RiPPs share a common biosynthesis pathway consisting of the mRNA-dependent synthesis of a precursor peptide that undergoes post-translational modifications (PTMs) on a core sequence before being excised from a leader sequence, the PTMs carried by RiPPs and their structures are highly diverse, thus determining their classification [12,13].

Recently, we have characterized such a RiPP, the ruminococcin C1 (RumC1), produced by the bacterial strain E1 of one of the prominent members of the human gut microbiome, *Ruminococcus gnavus* [14,15,16]. We previously demonstrated that RumC1 belongs to the sactipeptide subclass, as it carries four sulfur to α–carbon thioether cross-links, leading to a hitherto undescribed and highly compact three-dimensional structure, which confers a high resistance to the physiological conditions encountered during systemic or oral administration [15,17]. RumC1 displays potent in vitro activity against Gram-positive pathogens, including clinical isolates and MDR strains [15,17]. Moreover, RumC1 retains its activity in the presence of a simulated and infected intestinal epithelium and was shown to be safe both in vitro on human cell lines and ex vivo on human intestinal tissues [15,17]. To our knowledge, this original sactipeptide possesses more clinical properties than any other described members of this subclass of RiPPs.

Taking into account all the promising clinical properties of RumC1, we decided to take this study here a step further in order to validate the potency of RumC1 as a drug-lead in vivo on an infected animal model. In addition, we evaluated the ability of RumC1 to kill a clinical isolate of *C. perfringens* in a complex microbial intestinal environment, while studying its impact on the microbiome distribution. Finally, we expanded our investigation on the other biological properties typical of the RiPP family, such as anti-biofilm, antifungal, or anti-inflammatory activities, which could implement the consideration of RumC1 as a drug-lead.

## 2. Results

### 2.1. RumC1 Is Effective to Clear a C. perfringens Infection In Vivo

**Figure 1:**
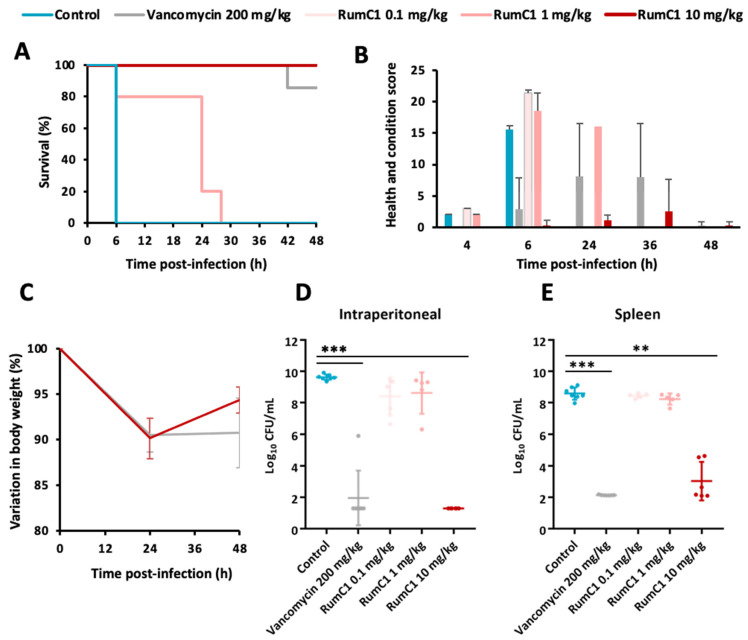
In vivo antibacterial efficacy of RumC1. Mice were challenged intraperitoneally with *C. perfringens* CP24. After 0.5, 1, and 4 h post-infection (hpi), vancomycin and RumC1 were injected intraperitoneally at 200 mg/kg and 0.1, 1, or 10 mg/kg, respectively, or PBS was injected (control). The groups were composed of five to seven animals. (**A**) The survival of mice was followed over 48 h. The survival of mice that received RumC1 at 0.1 mg/kg does not appear on the figure as their survival had the same evolution as the control mice. (**B**) The health and physical condition of mice was measured over time. High scores are representative of impaired health and physical condition. Criteria measured and observed to determine this score are listed on Figure S2 (**C**). The weight of mice that were still alive at 24 and 48 hpi was measured. (**D**,**E**) *C. perfringens* loads found in the intraperitoneal cavity (**D**) or in the spleen (**E**) were measured by plating at the time of death or at the end of the study for the surviving mice (i.e., 48 hpi). Normally distributed data were analyzed using analysis of variance to compare the effects between the different groups, followed by a Bonferroni test to compare the treated groups two by two (** *p*-value < 0.01, *** *p*-value < 0.001).

Previously, we showed that RumC1 is active against Gram-positive pathogens in vitro, on laboratory strains as well as on clinical isolates [15,17]. In particular, RumC1 is active under the micromolar range against multiple strains of *C. perfringens* with minimum inhibitory concentrations (MICs) similar to the conventional antibiotic vancomycin [17], commonly used for the treatment of gut infections caused by Clostridia. Here, we evaluated and compared the efficacy of RumC1 and vancomycin to protect mice from a *C. perfringens* infection. Mice were infected with the clinical isolate *C. perfringens* CP24 by intraperitoneal injection and then received either phosphate-buffered saline (PBS), vancomycin, or RumC1 delivered intraperitoneally at 0.5, 1, and 4 h post-infection (hpi). In the control group that received PBS, the infection was lethal for all the mice at 6 hpi (Figure 1A). Since preliminary studies revealed that vancomycin administration at 110 mg/kg led to mice dying starting at 24 hpi (Figure S1), it was delivered at a higher dose of 200 mg/kg for each injection. To evaluate the efficiency of the sactipeptide, three groups of mice treated with RumC1 were constituted with doses of 0.1, 1, and 10 mg/kg, injected three times. Strikingly, RumC1 at 10 mg/kg was sufficient to protect 100% of the infected mice, whereas similar treatment with vancomycin at 200 mg/kg led to the survival of only 85% (Figure 1A). On the contrary, RumC1 at 0.1 mg/kg had no impact on the survival of mice, whereas no mice treated with 1 mg/kg survived longer than 28 hpi.

Moreover, between the two groups that survived until the end of the study (48 hpi), i.e., mice treated with either RumC1 at 10 mg/kg or vancomycin at 200 mg/kg, mice that received RumC1 exhibited the lowest scores of impaired health and physical condition throughout the experiment, although the scores increased in both cases during the infectious phase (Figure 1B and Figure S2). Notably, the impaired health and physical condition score variations over time associated with RumC1 treatment at 10 mg/kg were lower than the variations associated with vancomycin treatment. In both cases, the weight of the mice dropped by almost 10% at 24 hpi. Treatment with RumC1 at 10 mg/kg led to a weight restoration of 5.7% at 48 hpi, whereas mice treated with vancomycin at 200 mg/kg did not gain their weight back at the end of the study (Figure 1C). Besides the surveillance of the overall physical condition of the animals, we performed a blood analysis (Figure S3A). The untreated mice challenged with *C. perfringens*, displayed high hemoglobin concentrations and elevated hematocrit, resulting in polycythemia, and also suffered from thrombocytosis. Treatments with both RumC1 at 10 mg/kg and vancomycin at 200 mg/kg were successful in restoring standard values of blood cells and hemoglobin concentrations. The condition of thrombocytosis improved with both treatments although the thrombocyte concentrations remained slightly high in regard to the standard values. Finally, the concentration in serum of IL-6, a pro-inflammatory cytokine used as a marker of sepsis [18], was quantified (Figure S3B). Treatments with RumC1 at 10 mg/kg and vancomycin at 200 mg/kg resulted in at least a 17-fold decrease compared to untreated and infected mice.

Bacterial loads of *C. perfringens* contained in the peritoneal cavity or in the spleen of the mice at the time of death were determined by plating and colonies counting (Figure 1D,E). The mice treated with the low doses of RumC1, i.e., 0.1 or 1 mg/kg, displayed reduced contents of *C. perfringens* in the peritoneal cavity compared to control mice, but barely any reduction in the spleen. On the contrary, no *C. perfringens* was detected in the peritoneal cavity of mice that received the high dose of RumC1 of 10 mg/kg, and the loads of the pathogens were drastically reduced in the spleen. Regarding now the treatment with vancomycin, no *C. perfringens* was detected in the spleen, but one out of seven mice still had high bacterial contents in the peritoneal cavity. Overall, taking into account the survival, weight, and health of mice, as well as the bacterial cell counts, RumC1 was at least as efficient as vancomycin at curing mice from a *C. perfringens* infection, but at a much lower dose.

### 2.2. RumC1 Effectively Kills C. perfringens in a Complex Microbial Community

**Figure 2:**
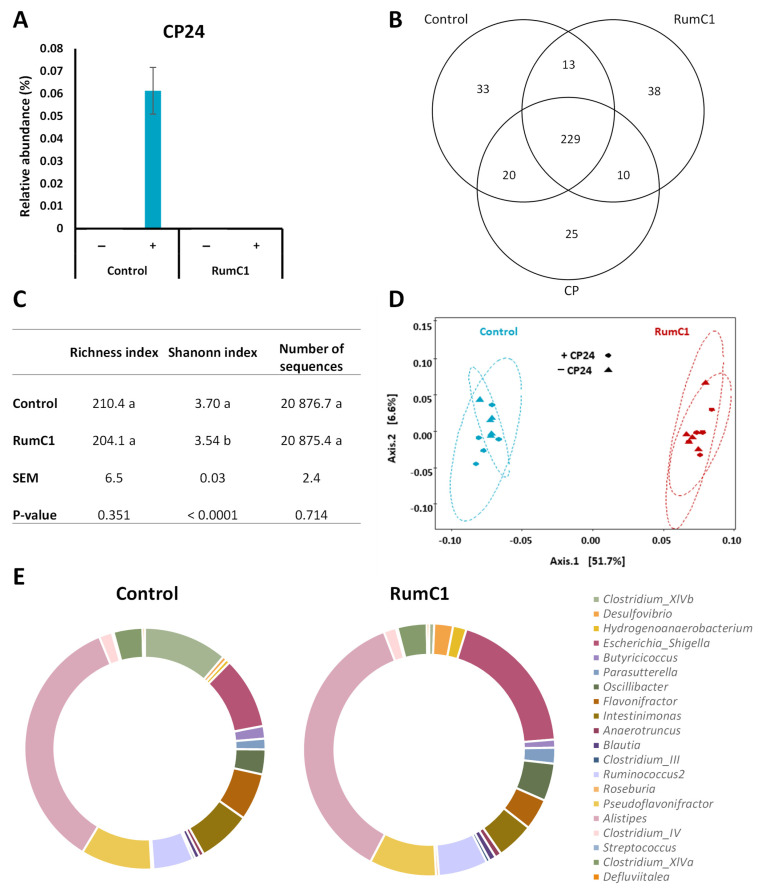
Clearance of *C. perfringens* by RumC1 in a complex microbial community. The cecal contents of broiler chickens were supplemented with *C. perfringens* CP24 at 106 CFU/mL (+), or not (−), and then with RumC1 at 5 × MIC of *C. perfringens* CP24 or left untreated (control). Microbial fermentation was then performed in anaerobic conditions for 24 h at 39 °C. The composition of the microbiota from each condition was obtained by 16S rRNA sequencing. Each treatment was done on five replicates. (**A**) Detection of the 16S rRNA gene sequence of CP24 and its relative abundance in the microbial community. (**B**) Venn diagram representing the number of shared or unshared ASVs between the control group (neither CP24 nor RumC1), the cecal contents inoculated with CP24 (group CP, no RumC1), and the cecal contents supplemented with RumC1 (group RumC1, no CP24). (**C**) Diversity index table of the control and RumC1 groups shown in (**B**), SEM = standard error of the mean; a, b: different letters denote significant differences. (**D**) Principal coordinate analysis of the untreated and RumC1-treated cecal contents inoculated with CP24 or not. % in axis shows the degree of explanation (axis 1 explains 51.7% of the variance). (**E**) Major genera and clusters with relative abundance >0.1% composing the microbial communities of untreated and RumC1-treated cecal contents. Inoculation of CP24 does not affect these distributions in each group (control vs. RumC1).

After showing that RumC1 was efficient at protecting mice from a lethal *C. perfringens* infection, and taking into account that RumC1 is active against a broad spectrum of Gram-positive bacteria [15,17], we wanted to investigate if RumC1 could effectively kill *C. perfringens* in the context of a complex and diverse natural microbiome. As *C. perfringens* CP24 was isolated from a broiler chicken [19], we used the corresponding cecal contents as the source of intestinal microbiome. These cecal contents were either supplemented with *C. perfringens* CP24 or not, and then treated with RumC1 at a dose equivalent to 5 × MIC (MIC = 1.56 µM in brain–heart infusion media) on this clinical isolate, before being incubated anaerobically at 39 °C for 24 h. The composition of the microbiome was then determined by 16S rRNA sequencing. A sequence corresponding to the 16S rRNA gene of *C. perfringens* CP24, previously sequenced, was detected only in the cecal contents supplemented with this strain in the absence of RumC1 at a relative abundance of 0.06% (Figure 2A). Interestingly, RumC1 efficiently inhibits the colonization of a natural intestinal chicken microbiome by *C. perfringens* CP24.

### 2.3. RumC1 Favorably Modulates the Intestinal Bacterial Ecology

**Figure 3:**
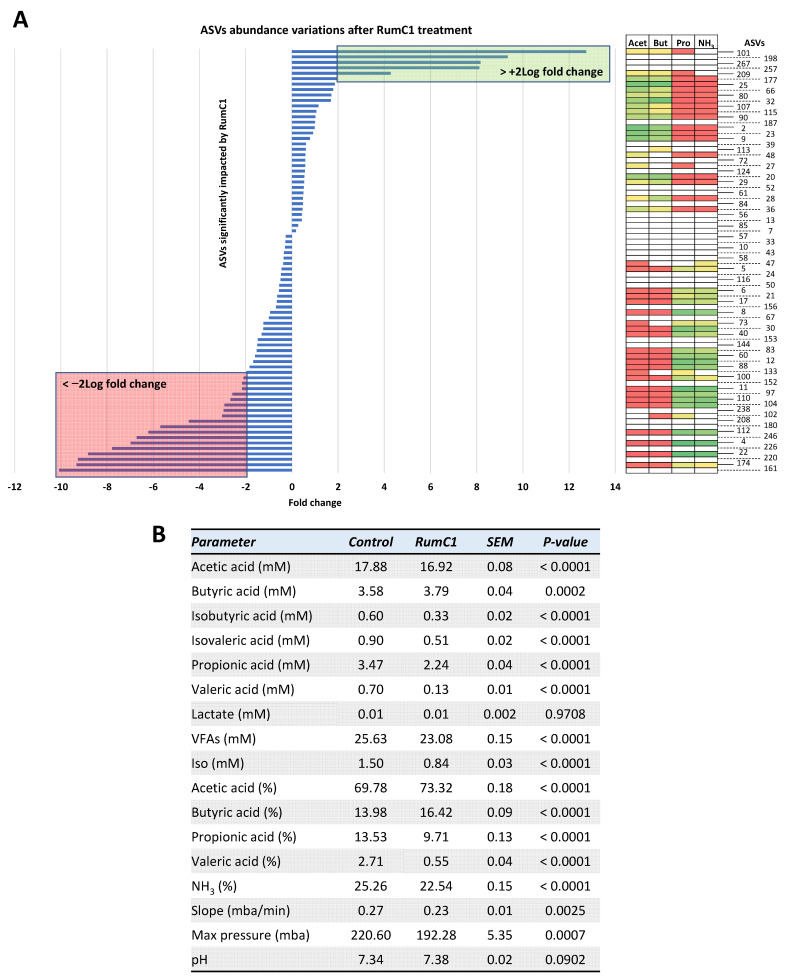
Main ASVs impacted by RumC1 treatment and their correlation with SCFA production. Cecal contents of broiler chickens were supplemented with *C. perfringens* CP24 at 10^6^ CFU/mL and treated with RumC1 at 5 × MIC of *C. perfringens* CP24 or left untreated (control). (**A**) The ASVs significantly (i.e., Pearson > 0.8 and *p*-value < 0.05) impacted by RumC1 are shown on the figure as well as their correlation with metabolite production using a color gradient from red (poor producer) to green (good producer), respectively. (**B**) SCFA production and fermentation parameters for each group were statistically analyzed by ANOVA test. SEM = standard error of the mean. Slope, maximum pressure, and pH were the parameters measured during the fermentation. Slope = rate of gas production in the exponential phase, max pressure = maximum gas production in 24 h, pH = final pH after 24 h of fermentation.

Subsequently, to demonstrate that RumC1 is able to kill *C. perfringens* in a complex intestinal bacterial microbiota, we analyzed its impact on the rest of the microbiome. Out of the 368 amplicon sequence variants (ASVs) detected in the dataset, 295, 290, and 284 ASVs were detected in the untreated, RumC1-treated (at 5 × MIC) and *C. perfringens* CP24-inoculated chicken cecal contents, respectively. More than 80% of the ASVs were shared between the untreated and RumC1-treated groups (Figure 2B). Treatment of the chicken cecal contents with RumC1 resulted in a decrease in the Shanonn index, revealing that the evenness was significantly impacted by RumC1. However, the richness was identical between the RumC1-treated and untreated cecal microbiota (Figure 2C). The general structure of the bacterial community was also sensitively but significantly impacted by RumC1, whether *C. perfringens* CP24 was added to the cecal contents or not (Figure 2D). Overall, these results suggest that RumC1 modulates the bacterial population without major disappearance of bacterial species. In particular, in cecal contents supplemented with CP24, statistical analysis at the ASV level showed that 78 out of the 384 ASVs were significantly impacted by RumC1 addition (Figure 3A). Among those 78 ASVs, 44 were significantly inhibited, whereas 34 increased. Out of the most impacted ASVs (>2 log2 fold change, Figure 3A and Figure S4), the 17 negatively impacted ASVs were identified as Gram-positive, while 3 out of 5 positively impacted ASVs were identified as Gram-negative.

Interestingly, the relative abundance of the ASVs negatively impacted by RumC1 were correlated with high propionate/ammonia producer species, while the one increasing in the presence of RumC1 were correlated with high butyrate/acetate producers. Accordingly, RumC1 treatment, with or without the addition of CP24—which does not impact fermentation parameters—led to an increase in the proportion of butyrate and acetate in the bacterial community environment, while propionate, branched short-chain fatty acids (SCFAs) and ammonia decreased (Figure 3B). However, overall, only a slight decrease in SCFA concentration was observed (23.1 mM vs. 25.7 mM for the RumC1-treated and untreated groups, respectively). The impact of RumC1 on the proportion of SCFAs might create a beneficial gut environment. Indeed, high acetate/butyrate contents are often associated with a more fibrolytic microbial population [20], whereas the proportions of individual SCFAs usually change in favor of propionate in overweight and obese subjects [21]. Recently, butyrate has received particular attention for its beneficial effects on intestinal homeostasis and energy metabolism. Indeed, with anti-inflammatory properties, butyrate enhances intestinal barrier function and mucosal immunity [22].

Finally, we also analyzed the distribution of the microbiota according to the classification of the ASVs from phylum to genus taxonomic level (Figure 2E). The main differences consist of the major reduction in the *Clostridium* cluster XIVb from 8% of relative abundance to 0.5%, and a counterpart increase in Gram-negative bacteria, in particular *Escherichia* and *Shigella*. Additionally, Gram-negative *Desulfovibrio* and *Hydrogenoanaerobacterium* increased significantly in the RumC1-treated cecal contents but remained relatively low in abundance within the overall community. The species composing cluster XIVb of *Clostridium* were: *Anaerotignum propionicum* (formerly known as *Clostridium propionicum*), *Anaerotignum neopropionicum* (formerly known as *Clostridium neopropionicum*), *Anaerotignum lactatifermentans* (formerly known as *Clostridium lactatifermentans*) *Cellulosilyticum lentocellum* (formerly known as *Clostridium lentocellum*), *Cellulosilyticum ruminicola*, *Epulopiscium* sp., *Clostridium colinum*, and *Clostridium piliforme* [23,24,25]. These last two species are responsible for ulcerative enteritis in chickens and Tyzzer’s disease, respectively. In accordance, with the major decrease of species from the cluster of *Clostridium* XIVb, 5 out the 17 most negatively impacted ASVs correspond to strains of *Anaerotignum lactatifermentans* (Figure S4). Interestingly, the overall proportions of other *Clostridium* clusters, namely clusters IV and XIVa, were not impacted by RumC1, even though a few individual strains were either negatively or positively impacted by RumC1 (Figure 2E and Figure S4). These clusters were constituted by several genera and were mainly Gram-positive bacteria. In humans, these two clusters represent key players in gut homeostasis and can account for up to 10–40% of the total commensal community [25,26,27].

### 2.4. RumC1 Disrupts Biofilms

**Figure 4:**
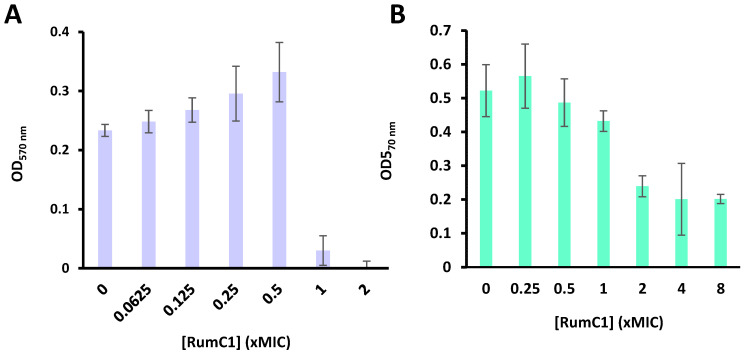
Anti-biofilm activity of RumC1. The inhibition of biofilm formation (**A**) or the disruption of the biofilm (**B**) was evaluated on *B. subtilis* ATCC 6633. This strain was grown in a Calgary biofilm device (CBD) in TSB (tryptic soy broth) for 48 h in the presence (**A**) or in the absence (**B**) of RumC1. (**B**) After 48 h of growth, the pegs from the CBD were transferred to a new 96-well plate containing fresh media supplemented with RumC1 and incubated for another additional 24 h. (**A**,**B**) Biofilm formation on the pegs from the CBD was evaluated with crystal violet staining as described in the Materials and Methods section. OD means Optical Density. Each experiment was repeated in independent triplicates.

In microbial populations, bacteria can exist in a planktonic mode of growth, meaning that they are isolated cells, or they can evolve in biofilm. In the latter, bacterial cells are adjacent and compose a complex community embedded in an extracellular matrix. It has been estimated that 80% of bacterial infections are caused by bacterial cells in a biofilm mode of growth. The extracellular matrix composed of DNA, exopolysaccharides, and proteins ensures the protection of bacterial cells, leading to a 10 to 1000-fold increase in bacterial resistance to antibiotics in the biofilm compared to their planktonic growth mode [28]. Therefore, the prevention of biofilm formation and the disruption of pre-formed biofilms are desirable traits for alternatives to antibiotics. Based on the previously demonstrated ability of RumC1 to kill *B. subtilis* ATCC 6633 with an MIC and a minimum bactericidal concentration of 0.4 µM [15], we assayed here the potency of RumC1 to prevent biofilms of this strain in Calgary biofilm devices (CBDs) [29]. On these devices, pegs are embedded in the lids of 96-well plates which allows the growth of a biofilm on the surface of the pegs soaking in bacterial cell suspensions. RumC1 was unable to prevent biofilm formation at concentrations lower than the MIC. At higher concentrations, biofilm formation was prevented as expected because the growth of planktonic cells in the suspension was also inhibited (Figure 4A). In a second assay, *B. subtilis* was grown in biofilm for 48 h before adding RumC1. Incubation with RumC1 for 24 h at 1 × MIC induced a slight biofilm disruption. Less than 50% of the pre-formed biofilm was left after incubation with RumC1 at 2 × MIC or higher concentrations (Figure 4B). Therefore, RumC1 does not have a preventive effect on biofilm formation at sub-inhibitory concentrations but is effective at degrading already formed biofilms.

### 2.5. RumC1 Exhibits a Selective Antifungal Activity

As many RiPPs exhibit several distinct biological activities, and in particular diverse antimicrobial effects [12], we investigated the antifungal activity of RumC1 on a panel composed of Ascomycota and Basidiomycota phytopathogens. No growth inhibition was detected on the strains of Ascomycota studied (i.e., MIC > 100 µM). The activity of RumC1 was assayed on two strains of Basidiomycetes, *Coniophora puteana* BFRM 497 and *Heterobasidion annosum* BRFM 524. Similar to the *Ascomycota* sp., no activity was detected against the first species of Basidiomycetes. However, the growth of *H. annosum* was inhibited in the presence of RumC1 at 12.5 µM (Table 1). *H. annosum* is one of the most studied forest fungi because this pathogen, responsible for conifer diseases, is the cause of the most important economic loss of the forest industry in the northern hemisphere [30,31]. Therefore, RumC1 seems to exert a highly selective antifungal activity.

### 2.6. RumC1 Has Beneficial Effects for the Human Host

**Figure 5:**
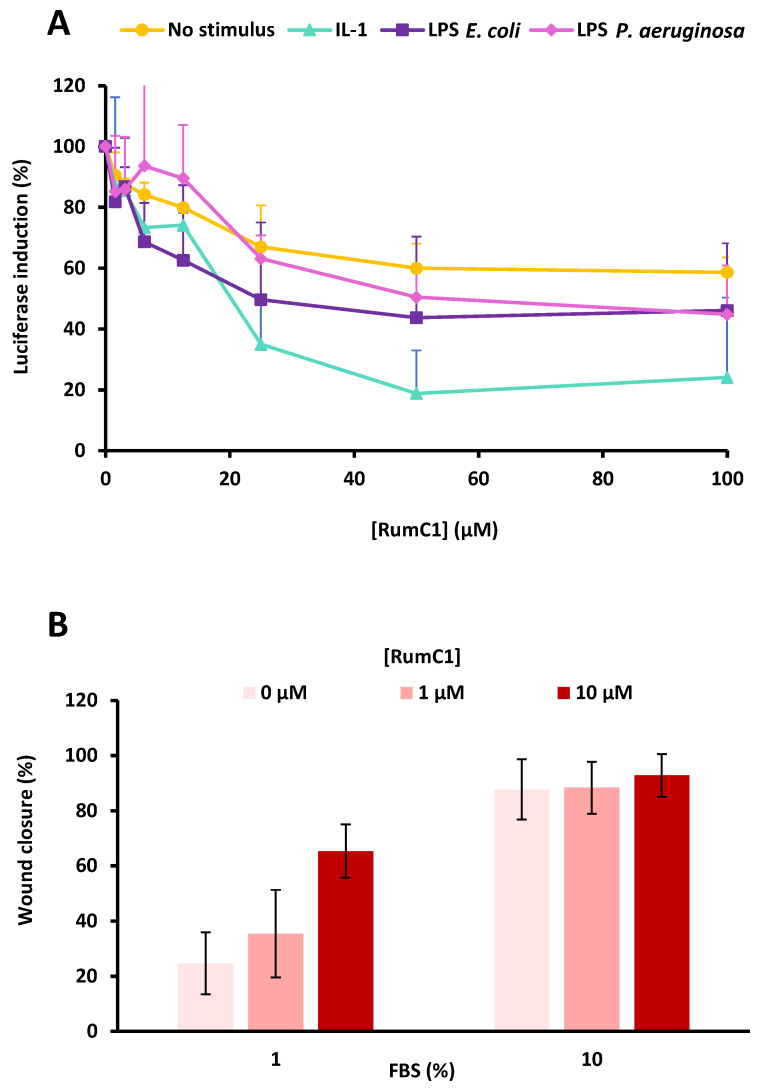
Host beneficial activities of RumC1. (**A**) Evaluation of the anti-inflammatory effect of RumC1. HeLa eLUCidate TLR4/IL-8 cells were incubated with IL-1β, LPS from *E. coli* or *P. aeruginosa*, or without any pro-inflammatory stimulus, and then treated with increasing concentrations of RumC1. Luciferase luminescence, induced by pro-inflammatory transcription factors, was measured. Results are expressed as the percentage of maximum response measured without RumC1. (**B**) Percentage of gap closure during a wound healing assay on HaCaT (human keratinocytes cells) cells in the absence or the presence of RumC1. Percentages of closure were measured four days after the gap formation and treatment. FBS (fetal bovine serum) at 10% was used as a positive control. All experiments were performed in independent triplicates.

Since RumC1 is produced by the human gut symbiont *R. gnavus* E1 [14,15,16], we also investigated the activities that RumC1 could display on the human host. First, the anti-inflammatory effect of RumC1 was assayed using the reporter cell line HeLa eLUCidate TLR4/IL-8. This cell line stably expresses at the membrane the Toll-like receptor (TLR) 4. TLRs are involved in the recognition of and response to bacterial infections, and TLR4 in particular is activated through binding to lipopolysaccharide (LPS). The activation of TLR4 induces the production of pro-inflammatory cytokines such as IL-1, IL-6, IL-8, IL-10, IL-12, and TNFα [32,33]. In the HeLa eLUCidate TLR4/IL-8 cell line, the expression of the *Renilla* luciferase reporter gene is under the control of the IL-8 promoter. Therefore, the detection of luciferase luminescence is directly linked to the induction of the inflammatory signaling mediated through LPS exposure. Here, we incubated this cell line with commercial LPS from *Escherichia coli* or *Pseudomonas aeruginosa*. Then, cells were treated with increasing concentrations of RumC1 or left untreated as controls, which were used to calculate the maxima of inflammatory responses. An inhibition of 50% of the inflammatory response was recorded with RumC1 at 25 and 50 µM, respectively, in the case of the induction by the LPS of *E. coli* and *P. aeruginosa* (Figure 5A). To discriminate between a general pro-inflammatory effect or a specific inhibition of the inflammation mediated by LPS, and thus by microbial infections, we also investigated the basal inflammatory status (in the absence of a pro-inflammatory stimulus) and the inflammatory response to IL-1β. IL-1β is another pro-inflammatory cytokine that causes the activation of the NF-κB signaling pathway, which regulates the transcription of many genes involved in inflammatory response, including IL-8 [34]. Without stimulus, the endogenous inflammatory response dropped by 40% in the presence of RumC1 at 50 µM. Moreover, at the same concentration, the inflammatory pathway stimulated by IL-1β was inhibited in the presence of RumC1 by 82% (Figure 5A). These results indicate, therefore, that RumC1 acts as a general anti-inflammatory molecule rather than a specific infection-associated anti-inflammatory molecule. Additionally, since the anti-inflammatory action of RumC1 is observed in the absence of stimulus as well as in the presence of endogenous (i.e., IL-1β) or exogenous (i.e., LPS) stimuli, RumC1 most likely acts directly on the eukaryotic cells, rather than interacting with the pro-inflammatory molecules used as stimuli.

Secondly, since some AMPs are able to stimulate tissue healing [35], we investigated the impact of RumC1 on wound healing. The human keratinocyte cell line HaCaT was grown in inserts, creating a gap within a cell monolayer. The insert was then removed, and cells were incubated with increasing concentrations of RumC1 before monitoring daily the migration of the cells to fill the gap. After four days of incubation in culture medium supplemented with 1% fetal bovine serum (FBS), untreated cells filled around 25% of the initial gap. HaCaT cells treated with RumC1 exhibited an increase in wound healing process in a dose-dependent manner. Indeed, cells treated with RumC1 filled the initial gap by 35 and 65% when incubated in the presence of 1 and 10 µM, respectively (Figure 5B, and Figure S5). As a positive control, HaCaT cells were incubated in culture medium supplemented with 10% FBS after gap creation. After four days, the gaps were filled by 88 to 93%, independently of RumC1 addition (Figure 5B and Figure S6). Thus, in conditions limiting cell growth, RumC1 speeds the migration and proliferation of this keratinocyte cell line and exhibits a positive impact on the wound healing process.

Finally, as some AMPs, and in particular RiPPs, have been shown to possess anticancer properties [12], we assayed the potency of RumC1 to inhibit the proliferation of cancer cell lines (i.e., pancreas and prostate human cancer cell lines); nevertheless, RumC1 does not seem to act as an anti-proliferative agent on the assayed cell lines as described in the supplementary information appendix (Figure S7).

## 3. Discussion

In this study, we clearly showed that RumC1 is active in vivo in an infected animal model. Considering the in vitro MIC of RumC1 and vancomycin against *C. perfringens* CP24 (1.56 µM and 0.8 µM in brain–heart infusion media, respectively) and taking into account the average peritoneal cavity volume, RumC1 was able to protect 100% of the mice challenged with *C. perfringens* at a dose corresponding to around 11 x MIC in vitro, whereas vancomycin only protected 85% of the mice at a corresponding dose of 1470 × MIC in vitro. Thus, RumC1 displays high potency against *C. perfringens* in vivo in a mammalian organism. The antibacterial potency of sactipeptides has been previously demonstrated only in vitro or, in the case of thuricin CD, ex vivo in a model of distal colon and in vivo on a transient and unlethal model of infection [36,37,38,39]. To our knowledge, this is the first time a sactipeptide has been shown to rescue an animal model from a lethal infection. In addition, we have also previously shown that RumC1 is safe for mammalian tissues. Here, we demonstrate at an effective antibacterial dose that an animal model tolerates RumC1 very well. Indeed, mice treated with RumC1 showed modest weight loss with the lowest scores in terms of impact on health and physical condition, rapidly restoring global blood constants and limiting the progression of inflammation, making it a good candidate for reaching the first clinical phase.

Although we demonstrated the high efficacy of RumC1 on a lethal mouse peritonitis model, it should be kept in mind that the major limitation of this widely used model lies in the absence of a complex microbial community in the peritoneal cavity. Thus, the potential interaction of RumC1 with commensal bacteria that could be deleterious for the efficacy of RumC1 against *C. perfringens* is not taken into account in this model. To consider RumC1 for the treatment of types of infections other than sepsis that occur in physiological compartments hosting microbial communities, such as the GI tract or the skin, for example, we studied the efficacy of RumC1 against *C. perfringens* in a complex microbial community using broiler chicken cecal contents. Though both models have their own drawbacks, they are complementary, as the first one allows for the study of the antibacterial activity of RumC1 in vivo and for taking into account its possible interactions with molecules and cells from a higher eukaryotic organism, whereas the second one considers the interactions with molecules and cells found in a whole microbiome. Strikingly, RumC1 was also able to kill *C. perfringens* in the complex chicken intestinal microbial community. While the rest of the community was partially affected by RumC1, being active against broad-spectrum Gram-positive bacteria, the overall distribution of the microbiome was only slightly disturbed. In particular, some major human intestinal commensal, including some Gram-positive bacteria, had the same distribution in the control and in the RumC1-treated microbiome. Moreover, we showed that the impact of RumC1 on the bacterial community might generate a favorable bacterial ecology in terms of SCFA production, likely to be associated with improved energy metabolism and anti-inflammatory properties. As we have previously shown that RumC1 (i) is naturally produced in the gut; (ii) is active against Gram-positive pathogens; (iii) is safe for human intestinal tissues; (iv) is active in the physiological gut conditions; (v) could be delivered orally [15,17]; is, as we have now demonstrated through this study, (vi) efficient at curing a microbial infection in an animal model; (vii) is effective at killing a pathogen colonizing an intestinal microbial community; (viii) has moderate impact on the latter community and especially on human commensal intestinal species; and finally, (ix) generates a favorable gut environment, RumC1 is most likely a well-suited alternative to antibiotics for the treatment of gastro-intestinal infections.

The human gut hosts a highly complex microbiota, and dysregulation of this community can lead to dysbiosis and trigger or worsen intestinal infections. In particular, antibiotic exposure can cause drastic changes in the microbial distribution and disrupt the gut barrier [40]. Therefore, antibiotics with a modest impact on the gut microbiome that could prevent dysbiosis would be highly useful. One of the major examples of this complex relationship between dysbiosis, bacterial infections, and antibiotics is the infection by *Clostridium difficile* (CDI). Extreme gut colonization by *C. difficile* can occur in individuals who suffer from dysbiosis, for example, after a heavy treatment with broad-spectrum antibiotics. As a result, CDIs are the most frequent nosocomial infections in the US [41]. Treatments with vancomycin, one of the most prescribed antibiotics for CDIs, often fail or result in relapses [42], and have been shown to provoke drastic alterations in microbiota and in particular to decrease commensal firmicutes and Gram-negative Bacteroidetes [37,43]. We have previously demonstrated the high in vitro potency of RumC1 against *C. difficile* [17]. As RumC1 seems to be safe for the dominant commensal species belonging to the *Clostridium* clusters IV and XIVa, further studies on the efficacy of RumC1 for the treatment of CDIs should be investigated.

Independently, RumC1 could be considered in fields other than bacterial infections. Indeed, we showed here that RumC1 also displays a highly selective antifungal activity against a forest pathogenic fungus. Of course, further in-depth studies of RumC1’s fungal spectrum could reveal if RumC1 could eliminate other fungi affecting humans, animals, or the environment. However, large-scale production investigation should be addressed before reaching such an objective.

In addition to its antibacterial activity, RumC1 displays a general anti-inflammatory effect, either directly, shown by the decrease of the inflammatory response of mammalian cells and in vivo on the infected mouse model by the decrease of the pro-inflammatory IL-6; or indirectly, by modulating SCFA production in the gut environment, which could be beneficial for the host, in particular in the context of a gastro-intestinal infections. Indeed, gut inflammation has been associated with several chronic inflammatory diseases, such as Crohn’s disease, that are often linked to dysbiosis, and can be triggered by several factors, including antibiotic exposure or infection [44,45,46]. Thus, antibacterial activity associated with an anti-inflammatory activity is a desirable trait for an alternative to antibiotics for the treatment of gastro-intestinal infections. RumC1, by inhibiting and killing Gram-positive bacteria, promotes the growth of *Escherichia* spp. by unlocking space for new ecological niches. As blooms of Enterobacteriaceae, including *Escherichia coli*, have been linked to inflammatory and chronic intestinal diseases, RumC1 could be misjudged as a pro-inflammatory molecule. However, and as described in the literature, these blooms of Enterobacteriaceae appear to be a consequence of inflammation, rather than a cause of inflammation in chronic intestinal diseases [44]. Consequently, the increase in *Escherichia* spp. proportions in the microbiota following RumC1 treatment is not a marker of intestinal inflammation that is very unlikely to occur, as RumC1 may actually help to reduce an inflammatory state in the host while clearing an intestinal infection. Finally, as seen before, the overall growth of *Clostridium* clusters IV and XIVa was not impacted by RumC1, and these clusters are well-known to be essential players in gut homeostasis [26,27].

Finally, as RumC1 displays a wide spectrum of action against Gram-positive pathogens and other clinical properties such as anti-biofilm or wound healing activities, infections occurring in other compartments could be considered as well. For example, we have previously shown that RumC1 displays high potency in vitro against *S. pneumoniae* [17], which is responsible for nasopharyngeal infections involving biofilm formation [47]. RumC1 could be a candidate for the in vivo clearance of infections caused by this pathogen. Moreover, antimicrobial molecules with wound healing properties are actively pursued, as non-healing wounds are often colonized by bacteria such as *E. faecalis*, another priority pathogen highly sensitive to RumC1 and well-known for its multidrug resistance [17,48]. It is estimated that 50% of chronic wounds, such as those encountered by people suffering from diabetes, are associated with bacterial biofilms. Molecules that promote skin cell migration and proliferation while inhibiting bacterial growth, especially in a biofilm mode of growth, and reducing inflammatory responses are attractive for wound healing therapies [48]. All those properties confirm the antimicrobial clinical potential and highlights the multiple functional properties of ruminococcin C1, thus extending its therapeutic interest.

## 4. Materials and Methods

### 4.1. Animal Models

Six-week-old pathogen-free RjOrl:SWISS female mice (weight, 20–24 g) were obtained from Janvier Labs (ref SN-SWISS-F). These non-isogenic (outbred) mice, used frequently in bacterial infection models, reflect the heterogeneity of the mouse population better than inbred mice. Mice were housed in cages, given food and water ad libitum, and allowed to adapt to their new environment for 4 days before any procedures were initiated. All animals were kept in university-inspected and approved housing sites and maintained in specific-pathogen-free conditions (group-housed) at the UTE-IRS2 Nantes Biotech Animal Facility (UTE, Experimental Therapeutic Unit, Nantes, France). Littermates were randomly assigned to experimental groups. All authorizations for conducting experiments on animals have been obtained from the relevant authority (Prefecture des Pays de la Loire, agreement no. A44-279, APAFIS#10009-2017051509429559). The health status of the animals housed in the animal facility UTE is specific-pathogen-free (SPF, Federation of European Laboratory Animal Science Associations standards). This SPF status is controlled every 3 months using sentinel animals. All animal facility users are highly aware of the principles of the 3Rs (Replace, Reduce, Refine), thanks to the specific animal welfare program (SBEA).

Twelve fourteen-day-old broiler chickens (ROSS PM3), housed in cages and given food and water ad libitum, were euthanized, and their cecal contents were collected and pooled. All experiments were conducted according to the European Union Guidelines of Animal Care and the legislation governing the ethical treatment of animals, and investigators were certified by the French government to conduct animal experiments. The Center for Expertise and Research in Nutrition facilities are in accordance with agreement no. C 03 159 4 of the 6th of November 2008, relative to experimentation on vertebrate living animals (European regulation 24/11/86 86/609 CEE, ministerial decree of the 19th of April 1988).

### 4.2. Human Cell Lines

All cell lines used in this study are commercial: eLUCidateTM HeLa TLR4/IL8 cells were obtained from Genlantis (Cat#EL-IL8HELA), PC-3 (CRL-1435TM) and MIAPacA2 (CRL-1420TM) cells were purchased from ATCC, and HUVEC cells from Sigma-Aldrich (Darmstadt, Germany; CAT#200-05N). All cell lines were routinely grown on 75 cm^2^ flasks at 37 °C with 5% CO_2_ in DMEM supplemented with 10% FBS and 1% antibiotics.

### 4.3. Bacteria

*Clostridium perfringens* CP24, provided by University of Gent [19], was routinely grown in a Trexler-type anaerobic chamber in brain–heart infusion (BHI) media at 37 °C. When needed, *C. perfringens* CP24 was grown on the selective media CP ChromoSelect agar (Sigma-Aldrich). *Bacillus subtilis* ATCC 6633 was routinely grown in Luria–Bertani (LB) broth at 30 °C with agitation at 180 rpm.

### 4.4. Fungi

*Heterobasion annosum* BFR 524 and *Coniophora puteana* BFRFM 497 were obtained from CIRM-BRFM. *Aspergillus niger* ATCC 9142 was purchased from the ATCC collection. *Fusarium verticillioides* DSMZ 62264, *Stachybotrys chartarum* DSMZ 2144, *Microdochium bolleyi* DSM 62073, and *Penicillium verrucosum* DSM 12639 were all purchased from DSMZ. All strains were grown on potato dextrose (PD) agar, at 25 °C.

### 4.5. In Vivo Efficacy of RumC1

Immunocompetent mice were infected intraperitoneally with 600 µL of appropriately diluted cell suspensions corresponding to the LD100 for *C. perfringens* CP24 isolate. Drugs were prepared in sterile PBS and administered by intraperitoneal injections (200 µL) at 0.5, 1, and 4 hpi. Animals were randomly assigned to either no treatment (control, PBS, n = 7); vancomycin at 200 mg/kg (n = 7); or RumC1 at 0.1 mg/kg (n = 5), 1 mg/kg (n = 5), or 10 mg/kg (n = 6). Survival rates were recorded at 1, 4, 6, and 24 hpi, and three times daily on subsequent days until the end of the 2 day observation period. Percentage body weight change and the scoring system for the assessment of disease severity (according to the criteria listed in Figure S2) for each animal was recorded daily. All surviving mice were euthanized at 48 hpi. For each animal, the spleen was removed, weighed, and homogenized in 1 mL of saline buffer (Mixer Mill MM400, RETSCH, Eragny sur Oise, France) and peritoneal fluid (5 × 1 mL, PBS) was collected. Both the spleen homogenates and the peritoneal fluids were used for quantitative cultures on *C. perfringens* selective media CP ChromoSelect agar (Sigma-Aldrich) for 24 h at 37 °C under anaerobic conditions. Viable counts were expressed as the mean (±SD) log_10_CFU per gram of organ or per mL of peritoneal fluids. Blood was collected for IL-6 quantification in heparin containing tubes, and for blood counts in EDTA (Ethylenediaminetetraacetic acid) containing tubes. Mice judged by experienced animal technicians to be experiencing pain or serious distress received buprenorphine (0.1 mg/kg, *s.c. b.i.d.,* sufficient to cover the nocturnal period) over the course of the experiment. Signs of unrelieved suffering triggered the humane endpoint of euthanasia by CO_2_ inhalation. Normally distributed data were analyzed using analysis of variance to compare the effects between the different groups, followed by a Bonferroni test to compare the treated groups 2 by 2 (GraphPad Prism Software, version 6.0).

### 4.6. Blood Analyses

Immediately after cardiac blood collection, the collecting tubes were filled through the straw of the container and stored and transported to the laboratory at a well-adapted and controlled cold temperature. Mice serum collecting tubes (EDTA) were then analyzed by fluorescence flow cytometry using a Sysmex XT-4000i hematology analyzer (Sysmex Europe GmbH, Norderstedt, Germany) and following standard procedure developed specifically for the automate. Blood cell composition in white blood cells, red blood cells, platelets, hemoglobin, and hematocrit were considered for this study. Whole blood was collected by cardiac puncture into heparin containing tubes and allowed to clot at room temperature for 30 min. After 10 min of centrifugation at 2000× *g* and 4 °C, the supernatant was transferred to a fresh polypropylene tube and immediately stored at −80 °C. Plasma were assayed for the presence of IL-6 using a Boster Picokine™ mouse IL-6 pre-coated ELISA (enzyme-linked immunosorbent assay) kit according to the manufacturer’s instructions (Boster Biological Technology, Pleasanton, CA, USA). This kit uses an ELISA based on a biotinylated antibody technique to assay mouse interleukin 6. Briefly, 100 µL of IL-6 standards and samples were added to the wells containing the capture antibody and incubated for 90 min at 37 °C. Biotinylated detection antibody was added, and the plates were incubated for 60 min at 37 °C. After 3 washes with PBS (10 mM, pH 7.2) bound detection antibody was then revealed by incubation (30 min at 37 °C) with avidin–biotin–peroxidase complex (ABC-HRP). The unbounded ABC-HRP was washed away with PBS before adding to 90 µL of TMB (3,3’,5,5’-Tétraméthylbenzidine) substrate. After 20 min of incubation in the dark, 100 µL of stop solution (H_2_SO_4_) was added, and 10 min later, the absorbance (OD) of each well was measured at 450 nm using a plate reader (Tecan, Männedorf, Switzerland). Concentrations of IL-6 in the samples were determined using standard curve draw using standard mice IL-6 solutions.

### 4.7. Fermentation of Chicken Cecal Contents

Cecal chicken microbial fermentation was performed in Hungate tubes in anaerobic buffer prepared as previously described [49]. The anaerobic buffer is composed of 5 solutions (A, B, C, D, and E) prepared individually: solution A (per liter: 5.7 g Na_2_HPO_4_, 6.2 g KH_2_PO_4_, and 0.6 g MgSO_4_-7H_2_O), solution B (per liter: 4 g NH_4_HCO_3_ and 35 g NaHCO_3_), solution C (per 10 mL: 132 mg CaCl_2_-2H_2_O, 100 mg MnCl_2_-4H_2_O, and 80 mg FeCl_3_-6H_2_O), solution D (per liter: 0.1% resazurine), and solution E (per 100 mL: 4 mL NaOH 1M, 625 mg Na_2_S). The anaerobic buffer was assembled under anaerobic conditions on the day of the experiment (using a mixture of CO_2_ and N_2_): 0.01% of solution A, 25.3% of solution B, 25.3% of solution C, 0.1% of solution D, 49.29% of ultra-pure water (18.2 mΩ), and autoclaved in the presence of 0.5 g/L of L-cystein (reducing agent). On the day of the experiment, the buffer was further reduced by adding 4% (*v*/*v*) of solution E, before adding the cecal inoculum (final pH: 7.5 and Eh: −150 mV). The cecal content was mixed (5% w/v) with the anaerobic buffer and 10 mL of the slurry was transferred into Hungate tubes. *C. perfringens* CP24 was inoculated in cecal fermentation medium at 10^6^ CFUs/mL. RumC1 was diluted in anaerobic buffer and added to the fermentation tube to obtain a final concentration of 5 × MIC of CP24, i.e., 7.8 µM. The fermentation was then performed in a water bath under constant agitation (200 rpm) at 39 °C for 24 h.

SCFA concentrations were analyzed by metaphosphoric acid extraction using gas chromatography and a flame ionization detector with 2-ethyl-butyric acid as an internal standard [50], NH_3_ was quantified using a Megazyme kit (K-AMIAR, Megazyme, Bray, Ireland), and lactate using a Thermo-Fisher kit (984306 and 84308, Thermo-Fisher). The difference in fermentation parameters between the groups was statistically analyzed by ANOVA test using R software (Miami, Florida, USA).

### 4.8. Taxonomic Analyses

Sequencing of 16 s RNA was done on an Illumina platform at GenoScreen using the Metabiote kit on the V3–V4 16 s hypervariable region. Briefly, DNA was extracted, normalized, and the multiplex library (30 samples using unique indexes) were prepared for the Illumina MiSeq paired-end sequencing, 2 × 300 bases. Quality control of the sequencing was performed using a mock community (15 bacterial and 2 archaeal strains), including in the sequencing run. The primer and index were identified (100% homology) and removed to create demultiplexed fastq files. The fastq files were quality trimmed at Q30 at the end of the read, the reads were then paired and assembled with a minimum 30 bp alignment at 97% homology using Qiime. The demultiplexed, quality trimmed, and assembled reads were then clustered using DADA2 software. The DADA2 package infers exact amplicon sequence variants (ASVs) from high-throughput amplicon sequencing data, replacing the coarser and less accurate operational taxonomic unit (OTU) clustering approach. The DADA2 pipeline takes demultiplexed fastq files as an input, and outputs the sequence variants and their sample-wise abundances after removing substitution and chimera errors. Taxonomic classification is done via a native implementation of the RDP naive Bayesian classifier. The normalized ASV table (normalized to the lower number of sequences/samples) is then analyzed using Phyloseq (Phyloseq objects containing ASV tables, taxonomic assignments, and environmental data), vegan and other bioconductor packages under an R environment to generate PcoA plots, diversity indexes, graphs, etc. Statistical analysis was performed using linear models, and the *p*-values were adjusted to account for multi-variable testing using the fdr (false discovery rate) method for the phylum and genera tables.

### 4.9. Antibiofilm Formation Assay

*B. subtilis* ATCC 6633 was grown in Luria–Bertani (LB) broth at 30 °C with agitation at 180 rpm until OD_600 nm_ reached 0.2–0.3. Cells were then diluted to 10^5^ CFU/mL in tryptic soy broth (TSB), and 150 µL of cell suspension was added per well of the Calgary biofilm device (CBD) [29]. Sterile RumC1 was added at a maximum concentration of 0.8 µM (i.e., 2 × MIC) and twofold series dilutions were performed in cell suspension. As it has been demonstrated that an edge effect affects the biofilm formation, all the edges of the plates were filled with TSB and used as sterility and negative controls [51]. After 48 h of incubation at 30 °C with agitation reduced to 110 rpm, the lid of the CBD was removed and the pegs were rinsed twice in PBS and then fixed in 100% methanol for 15 min. Pegs were rinsed with PBS once more and then air-dried before being stained with crystal violet at 0.2% for 15 min. Pegs were rinsed with PBS twice before being air-dried and then destained in methanol for 15 min before being discarded. Absorbance at 570 nm was measured to evaluate biofilm formation. All the rinsing, staining, and destaining steps were performed in 96-well plates with a volume of 200 µL per well in order for the biofilm to be totally soaked. All experiments were performed in independent triplicates, and measurements were acquired on at least two wells for each condition and for each replicate.

### 4.10. Antibiofilm Disruption Assay

A suspension of *B. subtilis* ATCC 6633 cells was prepared as described above, and CBD were filled with 150 µL of cell suspension without the addition of RumC1 and taking into account the edge effect previously mentioned. After 48 h of growth at 30 °C and under stirring at 110 rpm, the lid of the CBD was transferred into a new 96-well plate containing fresh TSB media supplemented either with RumC1 from 6.4 µM to 0.2 µM (8 × MIC and 0.25 × MIC, respectively) or not. Wells were filled with 200 µL to make sure the pegs were totally soaked. Then, after 24 h of incubation in the same condition, the pegs were rinsed, fixed, stained, and destained as described above. A volume of 300 µL was used per well. Absorbance at 570 nm was measured to evaluate biofilm formation and disruption. All experiments were performed in independent triplicates, and measurements were acquired on at least two wells for each condition and for each replicate.

### 4.11. Antifungal Assay

All targeted strains were grown on PD agar at 25 °C. Fungi suspensions were prepared by scraping spores with NaCl 0.85% + 100 µL/L of Tween 80 and were diluted to 2.10^4^ spores/mL in the appropriate broth (RPMI with MOPS, and PD for ascomycetes and basidiomycetes, respectively) after counting by microscopy with a calibrated cell. Sterile RumC1 was added to the fungi suspension in polypropylene 96-well microplates from 100 to 0.1 µM by twofold serial dilutions. Fungi were left for several days to grow at room temperature. MIC was defined as the lowest concentration of peptide inhibiting visible growth. Sterility and growth controls were included in each assay. MICs were determined in independent triplicates.

### 4.12. Evaluation of the Anti-Inflammatory Activity

Anti-inflammatory activity of RumC1 was evaluated using the commercial eLUCidate™ HeLa, TLR4/IL8 reporter cells (Genlantis, San Diego, CA, USA). This reporter cell line corresponds to stably transfected HeLa cells, which express human TLR4 (i.e., the receptor of LPS) as well as the *Renilla* luciferase reporter gene under the transcriptional control of the IL-8 promoter, making them a valuable model to detect the activation of IL-8 expression by LPS, but also by other stimuli causing NF-κB activation, such as IL-1. Cells were routinely grown on 75 cm^2^ flasks at 37 °C with 5% CO_2_ in DMEM supplemented with 10% FBS, 1% Pen/Strep, and selected antimitotic agents, i.e., puromycin (at 3 µg/mL), blasticidin (at 5 µg/mL), and G418 (at 500 µg/mL) (all from Sigma-Aldrich). To evaluate the anti-inflammatory effect of RumC1, eLUCidate™ HeLa cells were detached from 75 cm^2^ flasks using trypsin-EDTA solution (Thermo-Fisher), counted using a Malassez counting chamber, and seeded into 96-well cell culture plates at approximately 50,000 cells per well, following the manufacturer’s instructions. The next day, the wells were emptied and the cells were either left untreated (negative controls) or were treated with 100 µL of culture medium containing 10 ng/mL of LPS extracted from *P. aeruginosa* or *E. coli* (invivogen) or 10 ng/mL of human recombinant IL-1 beta (peprotech, Neuilly-Sur-Seine, France) in the presence of increasing concentrations of RumC1 (from 0 to 100 µM, serial 1:2 dilutions). Well-known anti-inflammatory molecules (i.e., pyrrolidine dithiocarbamate (PDTC) and epigallocatechin gallate (EGCG)), as well as a well-known blocker of LPS (i.e., polymyxin B), were used as positive controls (all from Sigma-Aldrich). Importantly, all steps were performed using non-pyrogenic plastics and RNase/DNase molecular biology tips to limit the risk of any presence of trace of LPS. After 6 h of incubation at 37 °C with 5% CO_2_, the wells were emptied, and the cells were lysed for 10 min at 4 °C with 70 µL of ice-cold PBS containing 1% Triton X-100. Fifty µL of cell lysates were then transferred into white 96-well luminescence plates already containing 100 µL of *Renilla* luciferase substrate (Yelen-Analytics, Marseille, France). Luminescence signals of the wells were immediately measured using a microplate reader (Biotek, Synergy Mx, Colmar, France).

### 4.13. Wound Healing Assay

The HaCaT keratinocyte cell line (obtained from Creative Bioarray) was cultured in Dulbecco’s modified Eagle’s medium (DMEM) supplemented with 10% FBS and 1% antibiotics (all from Thermofisher). Cells were routinely grown on 25 cm^2^ flasks and maintained in a 5% CO_2_ incubator at 37 °C in a 95% humidified atmosphere containing 5% CO_2_. Cells grown on 25 cm^2^ flasks were detached using trypsin-EDTA solution (Thermofisher, Illkirch-Graffenstaden, France). Cells were then diluted in culture medium and seeded into a silicone culture-insert 2 well in a 24-well plate (ibid.) developed for wound healing assay at approximately 200,000 cells per plate well. After cells reached confluence, the inserts were removed to create a gap, and the wells were washed 3 times with culture medium free of FBS and antibiotics. Cells were then treated with RumC1 or left untreated in culture medium supplemented with 1% FBS. Cells incubated with the culture medium supplemented with 10% FBS were used as a positive control. Nomarsky interference contrast images were acquired daily.

### 4.14. Antiproliferative Assay

Antiproliferative assays were performed on the human cancer cell lines PC-3 and MIAPaCa2, originating from human prostate and pancreatic cancer, respectively. The vascular endothelial primary cells, HUVEC (Human umbilical vein endothelial cells), were also included in the assay. PC-3 and MIAPaCa2 cells were cultured in Dulbecco’s modified Eagle’s medium (DMEM) supplemented with 10% heat-inactivated fetal calf serum and 1% antibiotics (all from Thermofisher), whereas HUVEC cells were grown in defined all-in-one ready-to-use endothelial cell growth medium (Sigma-Aldrich). Cells were routinely grown on 25 cm^2^ flasks and maintained in a 5% CO_2_ incubator at 37 °C in a 95% humidified atmosphere containing 5% CO_2_. The antiproliferative effect of RumC1 was evaluated as previously described [52]. Briefly, human normal and cancer cells grown on 25 cm_2_ flasks were detached using trypsin-EDTA solution (Thermofisher). Cells were diluted in the appropriate culture medium and seeded into 96-well cell culture plates (Greiner bio-one) at approximately 2000 cells per well. After 4 h to allow cell attachment, cells were then treated with increasing concentrations of RumC1 diluted in the appropriate medium. After 24 h, the medium was discarded and the number of viable cells was measured using resazurin assay as previously described [52].

### 4.15. Quantification and Statistical Analysis

Statistical details of experiments can be found in the figure legends and in the method details. All experiments were repeated at least three times. Data are presented as mean ± SEM in all figures. Statistical analyses were performed with GraphPad Prism 6.0 or R using the Bonferroni test, linear models with *p*-values adjusted to account for multivariable testing using the fdr method or an ANOVA test. In figures, *, **, and *** represent a significance defined as *p*-value < 0.05, < 0.01 and < 0.001, respectively.

## Figures and Tables

**Table 1:** In vitro antifungal activity of RumC1. Activity spectrum of RumC1 against selected Ascomycetes and Basiodiomycetes strains. MIC determination was performed in independent triplicates.

Division	Fungi	Strain	MIC (µM)
Basidiomycetes	*Heterobasidion annosum*	BRFM 524	12.5
	*Coniophora puteana*	BRFM 497	>100
Ascomycetes	*Aspergillus niger*	ATCC 9142	>100
	*Fusarium verticillioides*	DSMZ 62264	>100
	*Stachybotrys chartarum*	DSMZ 2144	>100
	*Microdochium bolleyi*	DSM 62073	>100
	*Penicillium verrucosum*	DSM 12639	>100

## Data Availability

The data presented in this study are available in the article and supplementary information file.

## Supplementary Materials

The following are available online at https://www.mdpi.com/1422-0067/22/6/3253/s1.
