# Supplementary material for: The Multifunctional Sactipeptide Ruminococcin C1 Displays Potent Antibacterial Activity In Vivo as Well as Other Beneficial Properties for Human Health
Source: Int J Mol Sci. 2021 Mar 23;22(6):3253. doi: 10.3390/ijms22063253 (PMC8005207; doi:10.3390/ijms22063253)
Supplement: Supplementary file 1 [file ijms-22-03253-s001.pdf]

The multifunctional sactipeptide Ruminococcin C1 displays potent antibacterial activity in vivo as well as other beneficial properties for human health

The SI file includes Figures S1 to S5 and Tables S1 to S3.

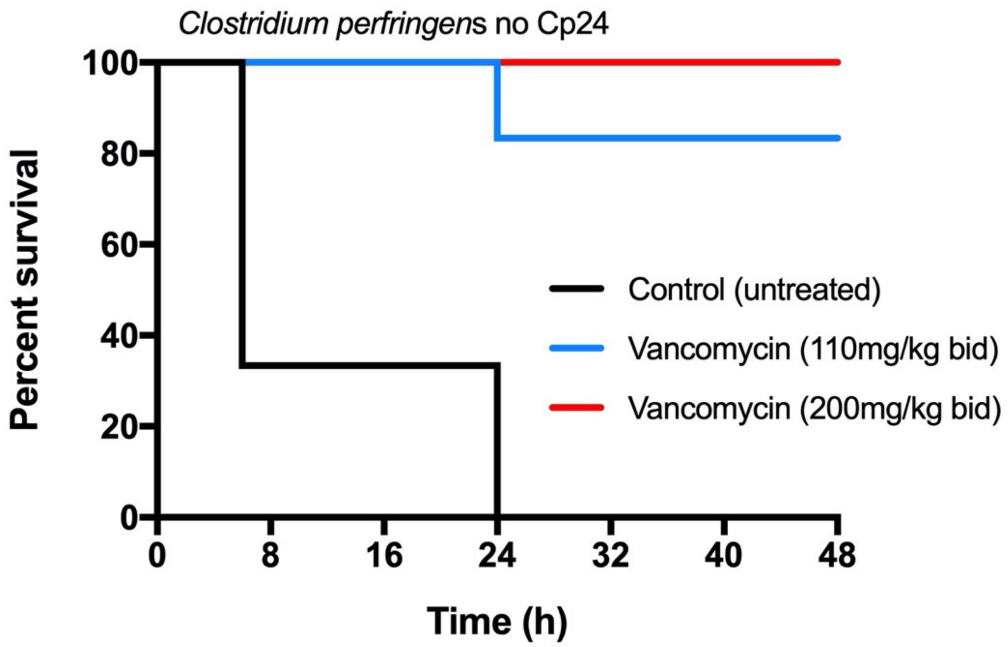

Figure S1. In vivo efficacy of vancomycin. In preliminary in vivo studies, mice were challenged with *C. perfringens* CP24 delivered by intra-peritoneal injection and treated with vancomycin injected in the peritoneal cavity twice daily (bid). Survival was followed.

A

Haemogram	*Reference Values*	*Control*	*RumC1 10 mg/kg*	*Vancomycin 200 mg/kg*
**Complete Blood Count**				
Leukocytes (G/L)	2.6-10.05	4.3 ± 0.8	4.9 ± 0.8	6.7 ± 0.7
Red blood cells (T/L)	6.5-10.1	**13.1 ± 3.3**	8.0 ± 0.4	8.6 ± 0.9
Haemoglobin (g/dL)	10.1-16.1	**21.1 ± 5.5**	12.9 ± 0.4	14.2 ± 1.2
Haematocrit (%. v/v)	32.8-48.0	**64.1 ± 15.8**	42.3 ± 1.7	45.1 ± 2.6
**Platelets**				
Trombocyte (G/L)	10-100	**595.8 ± 238.2**	**245.0 ± 15.5**	**208.0 ± 46.7**

B

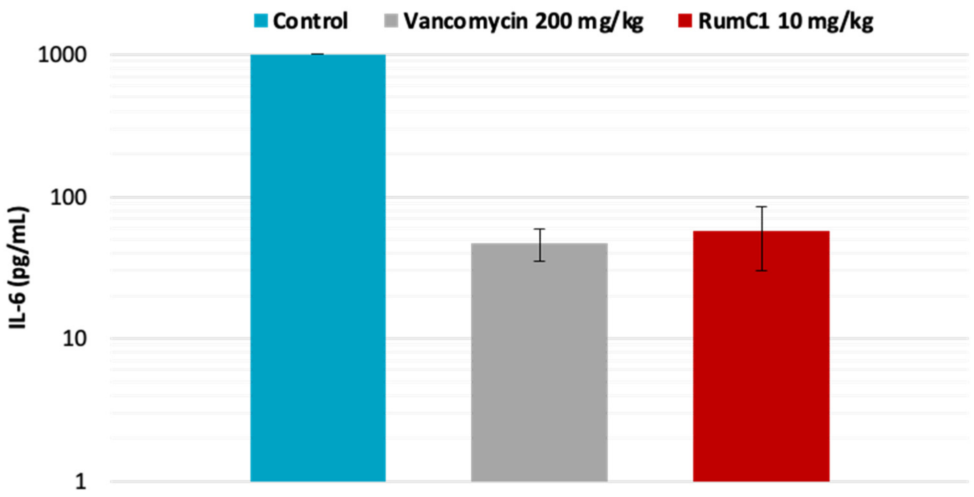

Figure S2. Blood analysis. The blood of mice infected with *C. perfringens* and untreated (control) or treated with either RumC1 at 10 mg/kg or vancomycin at 200 mg/kg was collected at the time of death. (**A**) The complete blood counts and platelets count were measured for each condition. Bold indicates values outside of the reference ranges. (**B**) IL-6 concentration in serum was measured for each condition.

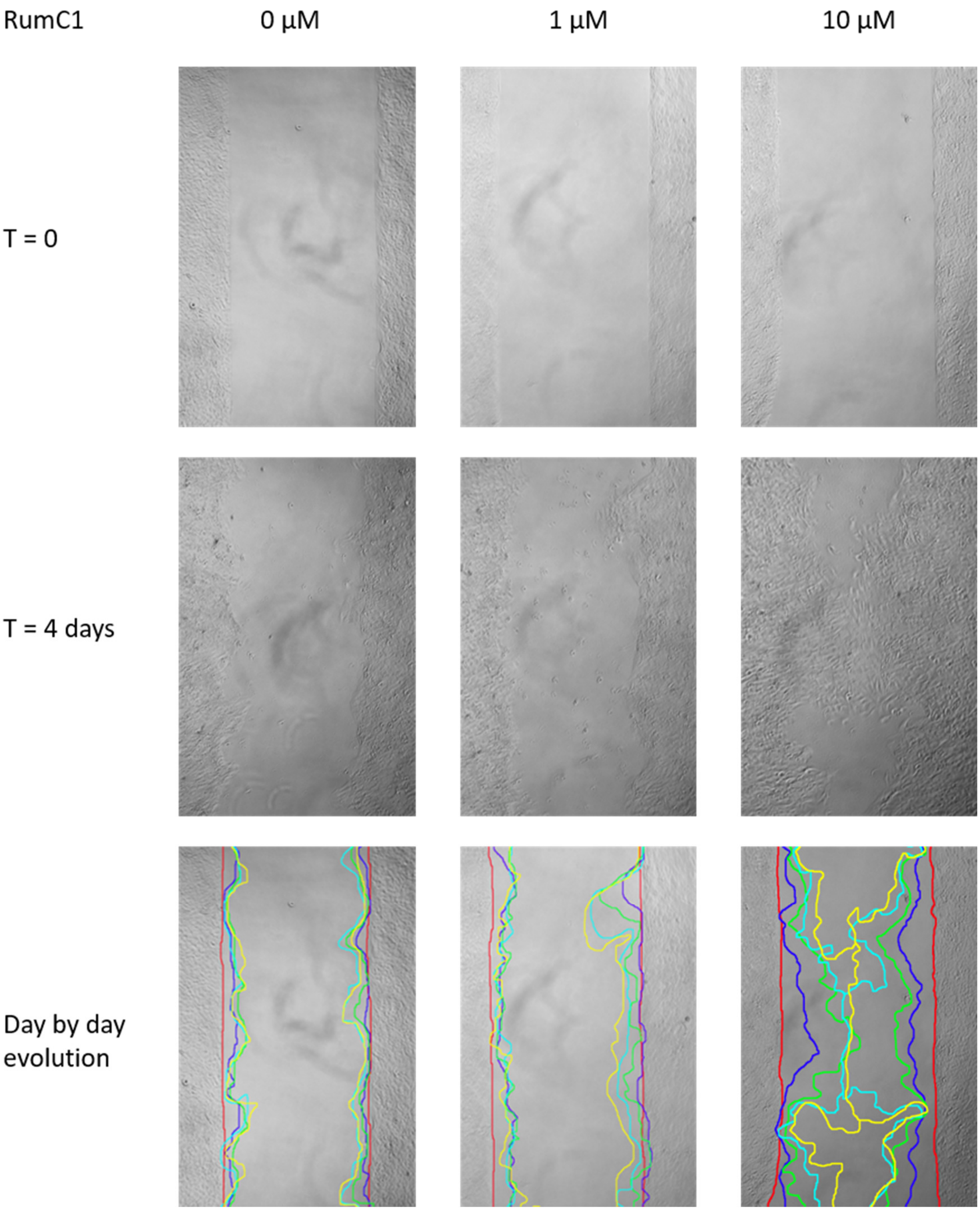

Figure S3. Migration of HaCaT cells in 1% FBS. On the first day of the experiment, a gap was formed in a HaCaT monolayer cell culture and cells were incubated with or without RumC1 in DMEM, FBS 1%. Gap closure was followed by microscopy daily and is represented by colored lines: red=day 0, dark blue=day 1, green= day 2, light blue= day 3, yellow=day 4.

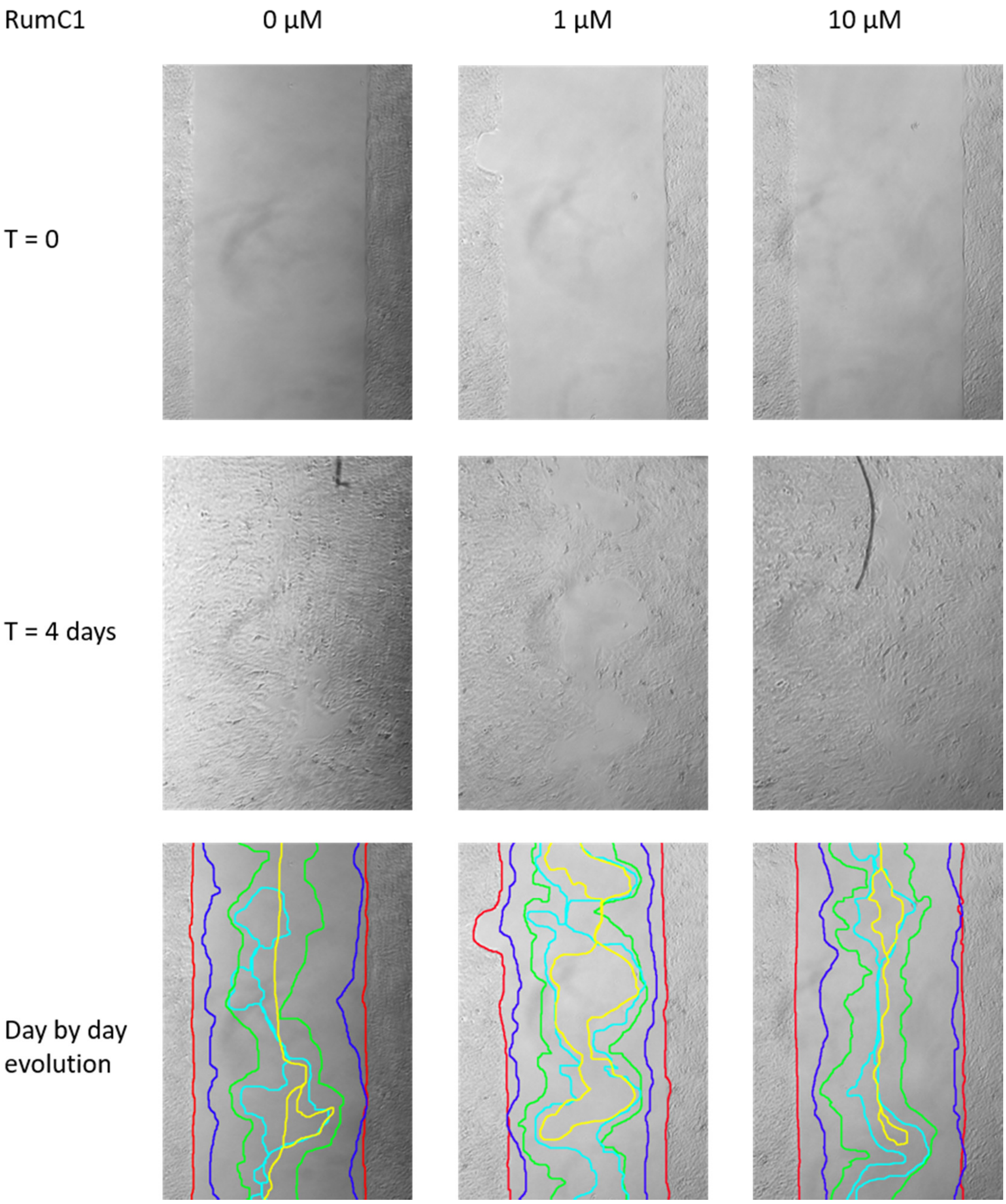

Figure S4. Migration of HaCaT cells in 10% FBS. On the first day of the experiment, a gap was formed in a HaCaT monolayer cell culture and cells were incubated with or without RumC1 in DMEM, FBS 10%. Gap closure was followed by microscopy daily and is represented by colored lines: red=day 0, dark blue=day 1, green= day 2, light blue= day 3, yellow=day 4.

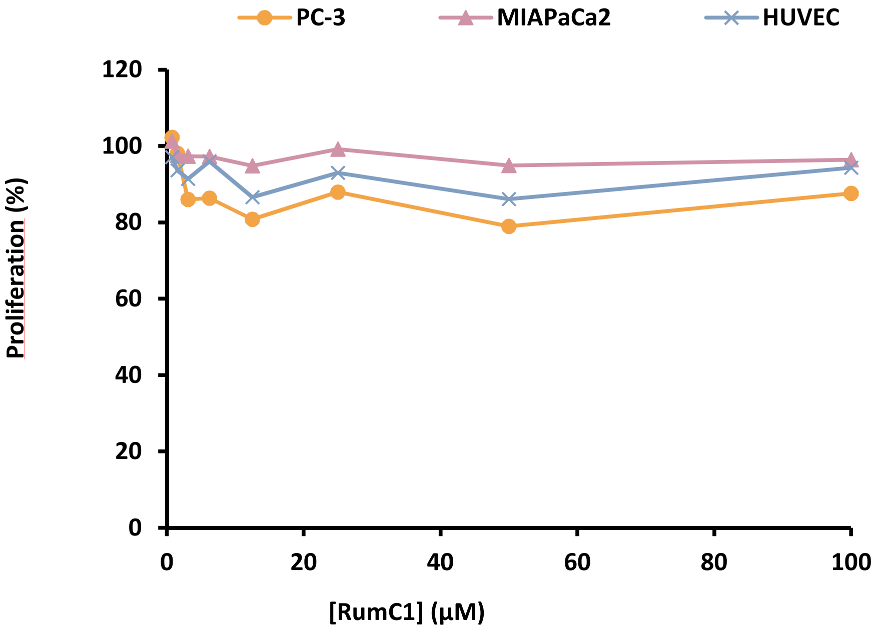

Figure S5. Antiproliferative assay. The cell lines PC-3 and MIAPaca2 as well as the primary vascular cells HUVEC were incubated with resazurin and conversion into fluorescent resorufin was monitored to determine their proliferation in the absence or presence of increasing concentration of RumC1. Results are expressed as the percentage of maximum response measured without RumC1.

We assayed the potency of RumC1 to inhibit the proliferation of cancer cell lines. Pancreas and prostate human cancer cell lines, respectively MIAPaCa2 and PC-3, were incubated with increasing concentration of RumC1 and their proliferation was followed. Endothelial HUVEC primary cells were also included in the assay, because of their high proliferative rates during angiogenesis. After 24 h of incubation with RumC1 a slight inhibition of proliferation was observed on the 3 cell models but under 10-20% of the maximum proliferation rate measured on untreated cells. Moreover, this low inhibition was not dose-dependent. Therefore, it seems that RumC1 does not act as anti-proliferative agent, at least on these specific human cell models.

Table S1. Health and physical condition score grid. The health of mice was monitored at 4 and 6 hours post-infection and then daily according to the criteria presented in this table. The sum of the scores of all the criteria give an overall health and physical condition score for each mouse Scores 0, 1, 2 corresponds to : no impact, medium impact and maximum impact respectively.

Localization	***Impact***	***Score***
Hair	Normal. stiff	0
	Bristly	1
General morphology	Normal	0
	Abdominal swelling	1
	Arched back	2
Weight Loss	< 10% of initial weight	0
	Between 10% and 20% of initial weight	1
	> 20% of initial weight	2
Eye lids	Opened	0
	Half-opened	1
	Closed	2
Tears	None	0
	Normal looking/red	1
	Eye glued/wounded	2
Mucosa/ears colors	Normal. pink	0
	Lighter color	1
	Yellow or blue	2
Aggressivity	None	0
	High (repeated biting)	1
Social behavior	United	0
	Isolated	1
Activities/games	Normal behavior	0
	Reduced activity	1
	Stereotypy	2
Breathing	Normal	0
	Faster or slower	1
	Loud/difficult/suffocation	2
**Total score**		

Table S2. Main ASVs impacted by RumC1. Caecal contents of broilers chickens were supplemented with *C. perfringens* CP24 at 10^6 CFU/mL and treated with RumC1 at 5xMIC of *C. perfringens* CP24 or left untreated (control). The table regroups all the ASVs impacted more than 2 log2 fold by RumC1 compared to control. The ASV in bold corresponds to *C. perfringens* CP24 introduced exogenously in the chicken cecal contents.

ASVs	*Log2 fold change with RumC1 (compared to control)*	*Mean abundance (%)*	*Genus*	*Gram*
ASV161	-10.08	0.03	*Intestinimonas*	+
ASV174	-9.33	0.02	*Clostridium_XlVb*	+
**ASV220**	**-9.26**	**0.02**	***Clostridium_sensu_stricto***	**+**
ASV22	-8.83	0.55	*Clostridium_XlVb*	+
ASV226	-7.78	0.01	NA	+
ASV4	-6.98	2.60	*Clostridium_XlVb*	+
ASV112	-6.22	0.05	*Intestinimonas*	+
ASV180	-5.70	0.01	*Hespellia*	+
ASV208	-4.46	0.01	*Clostridium_XlVa*	+
ASV102	-3.03	0.09	*Intestinimonas*	+
ASV238	-2.95	0.01	*Intestinimonas*	+
ASV104	-2.92	0.07	*Clostridium_IV*	+
ASV110	-2.66	0.07	*Oscillibacter*	-
ASV97	-2.58	0.09	NA	+
ASV11	-2.16	1.05	*Clostridium_XlVb*	+
ASV152	-2.15	0.03	*Intestinimonas*	+
ASV100	-2.10	0.08	*Clostridium_XlVb*	+
ASV209	4.29	0.02	*Clostridium_XlVa*	-
ASV257	8.12	0.01	*Anaerostipes*	+
ASV267	8.17	0.01	*Clostridium_XlVa*	-
ASV198	9.35	0.02	*Flavonifractor*	+
ASV101	12.74	0.17	*Desulfovibrio*	-

Table S3. Main ASVs impacted by RumC1. Caecal contents of broilers chickens were supplemented with *C. perfringens* CP24 at 10^6 CFU/mL and treated with RumC1 at 5xMIC of *C. perfringens* CP24 or left untreated (control). Grey highlights correspond to the match with an S_ab score >0.9 (i.e. fairly confidence that the match is correct at the species level).

	Best match RDP			
ASVs	***Species***	***Strain number***	***GenBank accession number***	***S_ab score***
ASV161	*Intestinimonas butyriciproducens*	SRB-521-5-I	KC311367	0.78
ASV174	*Anaerotignum lactatifermentans*	G17	AY033434	0.82
**ASV220**	***Clostridium perfringens***	**ATCC 13124**	**CP000246**	**1.00**
ASV22	*Anaerotignum lactatifermentans*	G17	AY033434	0.99
ASV226	*Catabacter hongkongensis*	HKU16	AY574991	0.65
ASV4	*Anaerotignum lactatifermentans*	G17	AY033434	1.00
ASV112	*Intestinimonas butyriciproducens*	SRB-521-5-I	KC311367	0.77
ASV180	*Faecalicatena orotica*	DSM 1287	FR749917	0.82
ASV208	*Anaerocolumna jejuensis*	HY-35-12	AY494606	0.84
ASV102	*Intestinimonas butyriciproducens*	SRB-521-5-I	KC311367	0.78
ASV238	*Intestinimonas butyriciproducens*	SRB-521-5-I	KC311367	0.78
ASV104	*Ruminococcus bromii*	ATCC 27255	L76600	0.63
ASV110	*Oscillibacter ruminantium*	GH1	JF750939	0.86
ASV97	*Merdimonas faecis*	BR31	KP966093	0.82
ASV11	*Anaerotignum aminivorans*	SH021	AB298756	0.79
ASV152	*Intestinimonas butyriciproducens*	SRB-521-5-I	KC311367	0.81
ASV100	*Anaerotignum lactatifermentans*	G17	AY033434	0.90
ASV209	*Enterocloster aldenensis*	RMA 9741	DQ279736	0.96
ASV257	*Anaerostipes butyraticus*	35-7	FJ947528	0.99
ASV267	*Acetivibrio ethanolgignens*	DSM 3005	FR749897	0.85
ASV198	*Flavonifractor plautii*	ATCC 29863	AY724678	0.97
ASV101	*Desulfovibrio piger*	ATCC29098	AF192152	0.95
